# Gender Dimorphism of the Cardiac Dysfunction in Murine Sepsis: Signalling Mechanisms and Age-Dependency

**DOI:** 10.1371/journal.pone.0100631

**Published:** 2014-06-19

**Authors:** Jianmin Chen, Fausto Chiazza, Massimo Collino, Nimesh S. A. Patel, Sina M. Coldewey, Christoph Thiemermann

**Affiliations:** 1 Queen Mary University of London, William Harvey Research Institute, Barts and the London School of Medicine and Dentistry, London, United Kingdom; 2 University of Turin, Department of Drug Science and Technology, Turin, Italy; 3 Jena University Hospital, Department of Anesthesiology and Intensive Care Medicine, Jena, German; Charite Universitätsmedizin Berlin, Germany

## Abstract

Development of cardiac dysfunction is associated with increased morbidity and mortality in patients with sepsis. Increasing evidence shows that gender determines the degree of inflammatory response of the host and that females tolerate sepsis better than males. It is unknown whether gender affects the cardiac dysfunction in animals or patients with sepsis. To investigate this, male or female C57BL/6 mice were subjected to either lipopolysaccharide (LPS)/peptidoglycan (PepG) co-administration or cecal ligation and puncture (CLP). At 18 hours after LPS/PepG injection or 24 hours after CLP, cardiac function was evaluated by echocardiography. The septic insult caused a significant cardiac dysfunction in both genders. However, the cardiac dysfunction was significantly less pronounced in females in comparison with males subjected to LPS (3 mg/kg)/PepG (0.1 mg/kg) or CLP. Compared with males injected with LPS (3 mg/kg)/PepG (0.1 mg/kg), western blotting analysis of the myocardium from females injected with LPS/PepG revealed i) profound increases in phosphorylation of Akt and eNOS; ii) significant decreases in phosphorylation of IκBα, nuclear translocation of the NF-κB subunit p65, decreased expression of iNOS and decreased synthesis of TNF-α and IL-6 in the heart. However, the gender dimorphism of the cardiac dysfunction secondary to LPS/PepG was not observed when higher doses of LPS (9 mg/kg)/PepG (1 mg/kg) were used. In conclusion, the cardiac dysfunction caused by sepsis was less pronounced in female than in male mice. The protection of female hearts against the dysfunction associated with sepsis is (at least in part) attributable to cardiac activation of the Akt/eNOS survival pathway, decreased activation of NF-κB, and decreased expression of iNOS, TNF-α and IL-6. It should be noted that the observed gender dimorphism of the cardiac dysfunction in sepsis was not seen when a very severe stimulus (high dose of LPS/PepG co-administration) was used to cause cardiac dysfunction.

## Introduction

Sepsis is one of the most common causes of morbidity and mortality among admissions to the intensive care unit [1], [2]. Sepsis is a systemic dysregulated hyperinflammatory and/or anti-inflammatory response to infectious stimuli, such as bacteria, viruses and fungi, which, when excessive, may progress to organ failure and death [3]. Development of myocardial dysfunction is associated with increased morbidity and mortality of sepsis. More than 40% cases of sepsis have cardiovascular impairment [4] and the presence of myocardial dysfunction can increase the mortality rate of affected patients to 70% [5].

There is now good evidence that gender is a key determinant in the degree of the host inflammatory response and even of outcome in patients with sepsis. In a number of clinical and epidemiological studies, a significantly increased survival rate was reported in female patients when compared with male patients with sepsis [6]–[9]. This may be associated with lower pro-inflammatory and higher anti-inflammatory cytokine levels in female patients [9]. Moreover, healthy female volunteers challenged with either lipopolysaccharide (LPS) or lipoteichoic acid (LTA) showed less pro-inflammatory response than males as demonstrated by lower levels of tumor necrosis factor (TNF)-α, interleukin (IL)-1β, IL-6 and IL-8 in blood [10]. In addition, severely injured male trauma-patients had a higher incidence of sepsis, multiple organ dysfunction syndrome and greater elevations in plasma procalcitonin and IL-6 compared with the equivalent group of females [11]. Further basic research studies also confirmed these clinical data on gender dimorphism following sepsis. These experimental studies suggested that females had immunologic advantage and showed a significantly increased survival rate compared with males following induction of polymicrobial sepsis by cecal ligation and puncture (CLP) [12]. In addition, estrogen treatment attenuated the liver dysfunction and intestine injury caused by sepsis in rats with decreased serum aspartate aminotransferase (AST), alanine aminotransferase (ALT) levels and ameliorated oxidative organ damage [13], while testosterone receptor blockade with flutamide following trauma-hemorrhage restored immune depression and significantly decreased the mortality after a subsequent septic challenge in male animals [14].

However, little is known about the impact of gender dimorphism on cardiac dysfunction caused by sepsis. Moreover, the mechanisms underlying the gender difference in susceptibility of the heart to a septic challenge are not understood. The present study was designed to determine whether the severity of myocardial dysfunction caused by either co-administration of LPS/peptidoglycan (PepG) or polymicrobial sepsis induced by CLP differs in male and female mice. Having found that the cardiac dysfunction associated with sepsis was less pronounced in female than in male mice, we have then investigated the potential signalling pathways that may have contributed to the observed differences.

## Materials and Methods

### Animals

The animal protocols followed in this study were approved by the Animal Welfare Ethics Review Board (AWERB) of Queen Mary University of London in accordance with the derivatives of both the Home Office guidance on the Operation of Animals (Scientific Procedures Act 1986) published by Her Majesty’s Stationery Office and the Guide for the Care and Use of Laboratory Animals of the National Research Council. All surgery was performed under ketamine/xylazine anesthesia and echocardiography was performed under inhalation anesthesia of isoflurane, buprenorphine was administered before surgery as well as 6 hours and 18 hours after surgery to reduce postoperative pain, and all efforts were made to minimize suffering of the animals. This study was carried out on ten week-old male (n = 29) and age-matched female (n = 22) C57BL/6 mice, weighing 20–30 g, and eight month-old male (n = 12) and age-matched female (n = 12) C57BL/6 mice (Charles River Laboratories UK Ltd., Kent, UK), weighing 35–50 g. The animals were allowed to acclimatize to laboratory conditions for a period of at least one week before any experimental procedures were initiated. They were housed in individually ventilated cages lined with an absorbent bedding material with no more than 6 mice per cage. The room temperature and humidity was maintained at 19°C–23°C and 55%, respectively. All animals had free access to a standard diet and water *ad libitum*. The feeding boxes were cleaned and disinfected every 3 days, and the water was changed on a daily basis to prevent infectious diseases. Animals were inspected for signs of illness and/or unusual behaviour by research staff at least once per day. All studies involving animals are reported in accordance with the ARRIVE guidelines for reporting experiments involving animals [15], [16].

### Model of LPS/PepG-induced Cardiac Dysfunction

Ten week-old male and female C57BL/6 mice received intraperitoneal administration of LPS/PepG (LPS; 3 mg/kg and PepG; 0.1 mg/kg or LPS; 9 mg/kg and PepG; 1 mg/kg in PBS; 5 ml/kg i.p.). Sham-treated mice were not subjected to LPS/PepG, but were otherwise treated the same way. Eighteen hours after LPS/PepG administration, cardiac function was assessed by echocardiography *in vivo*. Mice were then deeply anesthetized i.p. with ketamine/xylazine, and were killed by removing the hearts. Heart samples were stored at −80°C for further analyses. Mice were randomly allocated into eight different groups. The following groups were studied for the low dose LPS/PepG co-administration [LPS (3 mg/kg)/PepG (0.1 mg/kg)] study: (i) Male+vehicle (n = 6); (ii) Female+vehicle (n = 4); (iii) Male+LPS/PepG (n = 7); (iv) Female+LPS/PepG (n = 8). The following groups were studied for the high dose LPS/PepG co-administration [LPS (9 mg/kg)/PepG (1 mg/kg)] study: (i) Male+vehicle (n = 5); (ii) Female+vehicle (n = 4); (iii) Male+LPS/PepG (n = 11); (iv) Female+LPS/PepG (n = 6).

### Model of Polymicrobial Sepsis caused by Cecal Ligation and Puncture

Eight month-old male and female C57BL/6 mice were subjected to CLP. Sham-operated mice were not subjected to ligation or perforation of cecum but were otherwise treated the same way. We followed the original CLP protocol introduced by Wichterman and co-workers [17] with slight modifications including analgesia, antibiotic therapy and fluid resuscitation as described previously [18], [19]. Based on previous evidence and preliminary data, an 18-G needle was used with the double puncture technique in order to generate reproducible cardiac dysfunction during the early phase of sepsis (24 hours). Briefly, mice were anesthetized i.p. with ketamine (100 mg/kg) and xylazine (10 mg/kg) prepared in the same solution by using 1.5 ml/kg. Buprenorphine (0.05 mg/kg i.p.) was injected additionally to provide adequate analgesia. The rectal temperature of the animals was maintained at 37°C with a homeothermic blanket. The abdomen was opened via a 1.5 cm midline incision, and the cecum exposed. The cecum was ligated just below the ileocecal valve and punctured at both opposite ends. After a small amount of fecal matter was extruded from both ends, the cecum was placed back in its anatomical position and the abdomen was sutured. Ringer’s solution was given s.c. for resuscitation directly after surgery (1 ml/mouse) and 6 hours and 18 hours after surgery (0.5 ml/mouse). Antibiotic (Imipenem/Cilastin; 20 mg/kg s.c.) and analgesia (buprenorphine; 0.05 mg/kg i.p.) was administered 6 hours and 18 hours after surgery. At 24 hours after CLP, cardiac function was assessed by echocardiography *in vivo*. Mice were then deeply anesthetized i.p. with ketamine/xylazine, and were killed by removing the hearts. Heart samples were stored at −80°C for further analyses. Mice were randomly allocated into four different groups: (i) Male+sham-operation (n = 4); (ii) Female+sham-operation (n = 4); (iii) Male+CLP (n = 8); (iv) Female+CLP (n = 8).

### Assessment of Cardiac Function *in vivo*

Cardiac function was assessed in mice by echocardiography *in vivo* as reported previously [18], [19]. At 18 hours after LPS/PepG co-administration or 24 hours after CLP, anesthesia was induced with 3% isoflurane and maintained at 0.5 to 0.7% for the duration of the procedure. Before assessment of cardiac function, mice were allowed to stabilize for at least 10 minutes. During echocardiography the heart rate was obtained from ECG tracing and the temperature was monitored with a rectal thermometer. Two-dimensional and M-mode echocardiography images were recorded using a Vevo-770 imaging system (VisualSonics, Toronto, Ontario, Canada). Percentage fractional area change (FAC) was assessed from a two-dimensional trace and percentage EF and fractional shortening (FS) were calculated from the M-mode measurements in the parasternal short axis view at the level of the papillary muscles.

### Western Blot Analysis

Semi-quantitative western blot analyses were carried out in mouse heart tissues as described previously [20]. We assessed the degree of phosphorylation of Akt on Ser^473^, endothelial nitric oxide synthase (eNOS) on Ser^1177^, inhibitor of κB (IκB) α on Ser^32/36^, as well as the nuclear translocation of the p65 subunit of nuclear factor (NF)-κB (nucleus/cytosol ratio) and inducible nitric oxide synthase (iNOS) expression. Briefly, mouse heart samples were homogenized in 10% homogenization buffer and centrifuged at 4000 RPM for 5 minutes at 4°C. Supernatants were removed and centrifuged at 14 000 RPM at 4°C for 40 minutes to obtain the cytosolic fraction. The pelleted nuclei were re-suspended in extraction buffer and centrifuged at 14 000 RPM for 20 minutes at 4°C. The resulting supernatants containing nuclear proteins were carefully removed, and protein content was determined on both nuclear and cytosolic extracts using a bicinchoninic acid (BCA) protein assay following the manufacturer’s directions (Therma Fisher Scientific, Rockford, IL). Proteins were separated by 8% sodium dodecyl sulphatepolyacrylamide gel electrophoresis (SDS-PAGE) and transferred to a polyvinyldenedifluoride (PVDF) membrane, which was then incubated with a primary antibody (rabbit anti-total Akt, dilution 1:1000; mouse anti-pAkt Ser^473^, dilution 1:1000; rabbit anti-total eNOS, dilution 1:200; goat anti-peNOS Ser^1177^, dilution 1:200; mouse anti-total IκBα, dilution 1:1000; mouse anti-IκBα pSer^32/36^, dilution 1:1000; rabbit anti-NF-κΒ p65, dilution 1:1000; rabbit anti-total iNOS, dilution 1:200). Blots were then incubated with a secondary antibody conjugated with horseradish peroxidase (dilution 1:10000) for 30 minutes at room temperature and developed with the ECL detection system. The immunoreactive bands were visualized by autoradiography. Densitometric analysis of the bands was performed using the Gel Pro Analyzer 4.5, 2000 software (Media Cybernetics, Silver Spring, MD, USA). Each group was then adjusted against corresponding sham data to establish relative protein expression when compared with sham animals.

### Quantitative Determination of Tissue TNF-α and IL-6 by ELISA

The expressions of TNF-α and IL-6 in mouse heart samples were determined using mouse TNF-α and IL-6 immunoassay kits (R&D Systems, Minneapolis, MN), respectively, and have been normalized to the protein content.

### Statistics

All values described in the text and figures are presented as mean ± standard error of the mean (SEM) of n observations, where n represents the number of animals studied. Statistical analysis was performed using GraphPad Prism 6.0 (GraphPad Software, San Diego, California, USA). Two-way ANOVA followed by Sidak’s multiple comparisons test was used to compare intergroup differences. Comparing results were considered statistically significant when *P*<0.05.

### Materials

Unless otherwise stated, all compounds in this study were purchased from Sigma-Aldrich Company Ltd (Poole, Dorset, UK). All solutions were prepared using non-pyrogenic saline [0.9% (w/v) NaCl; Baxter Healthcare Ltd, Thetford, Norfolk, UK]. Antibodies for immunoblot analysis were purchased from Santa Cruz Biotechnology, Inc. (Heidelberg, Germany).

## Results

### Gender Dimorphism of Cardiac Dysfunction in Response to LPS (3 mg/kg)/PepG (0.1 mg/kg) Co-administration

**Figure 1:**
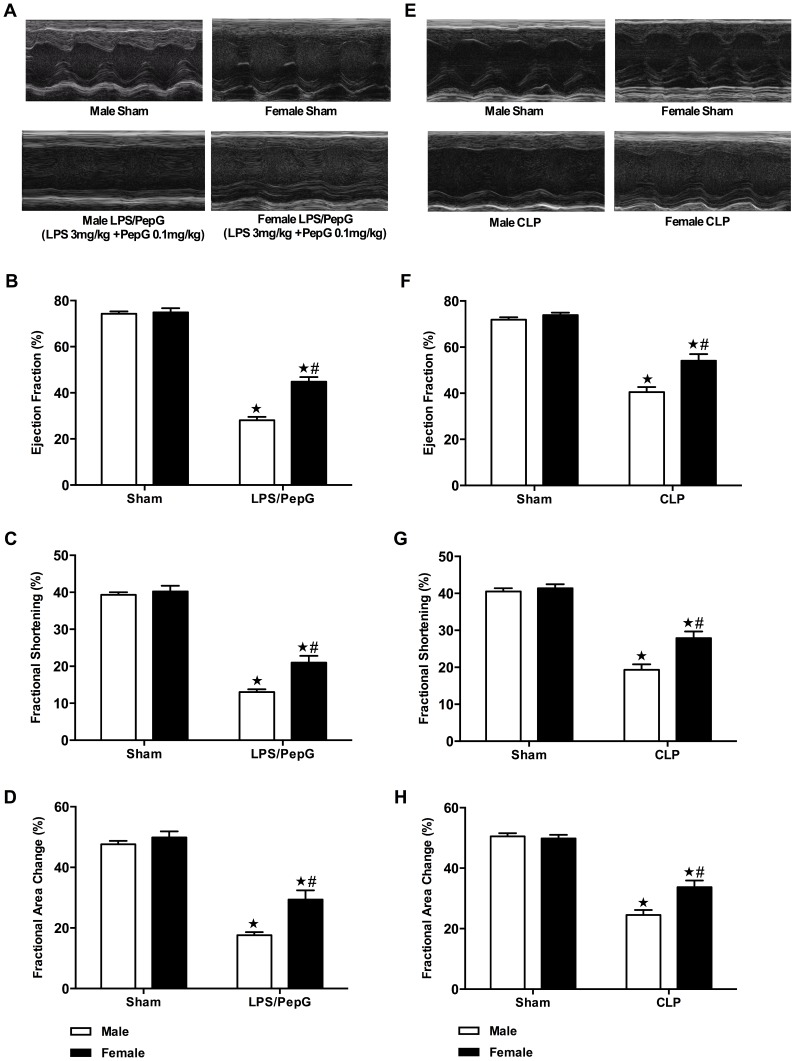
Gender dimorphism of cardiac dysfunction in mice subjected to LPS (3 mg/kg)/PepG (0.1 mg/kg) co-administration and in mice that underwent CLP. Panel **A**–**D**: Male or female mice received either LPS (3 mg/kg)/PepG (0.1 mg/kg) or PBS intraperitoneally. Cardiac function was assessed at 18 hours. (**A**) Representative M-mode echocardiograms; percentage (%) (**B**) ejection fraction (EF); (**C**) fractional shortening (FS); and (**D**) fractional area of change (FAC). The following groups were studied: Male+vehicle (n = 6); Female+vehicle (n = 4); Male+LPS/PepG (n = 7); Female+LPS/PepG (n = 8). Panel **E**–**H:** Male or female mice were subjected to CLP or sham-operation. Cardiac function was assessed at 24 hours. (**E**) Representative M-mode echocardiograms; % (**F**) EF; (**G**) FS; and (**H**) FAC. The following groups were studied: Male+sham-operation (n = 4); Female+sham-operation (n = 4); Male+CLP (n = 8); Female+CLP (n = 8). Panel **A**–**H**: Data are expressed as means ± SEM for n number of observations. ★*P*<0.05 versus the respective sham group, #*P*<0.05 versus male LPS/PepG or CLP group.

To determine the gender difference of cardiac dysfunction caused by LPS/PepG, left ventricular function was assessed using echocardiography at 18 hours after intraperitoneal injection of LPS (3 mg/kg)/PepG (0.1 mg/kg) or PBS (5 mg/kg). Mice injected with LPS/PepG had a lower body temperature and a lower heart rate in comparison to sham-treated mice (male sham/female sham versus male+LPS/PepG/female+LPS/PepG; *P*<0.05; Table 1). In sham-treated mice, there was no difference of EF, FS or FAC between male and female mice (*P*>0.05; Figure 1A–D). When compared to sham-treated mice, LPS/PepG caused a significant reduction in EF (*P*<0.05; Figure 1A–B), FS (*P*<0.05; Figure 1A, 1C) and FAC (*P*<0.05; Figure 1A, 1D) in both male and female mice, indicating the development of cardiac dysfunction *in vivo*. However, female mice subjected to LPS/PepG exhibited significantly higher EF, FS and FAC in comparison with male mice (*P*<0.05; Figure 1A–D), indicating the cardiac dysfunction caused by LPS/PepG was less pronounced in female than in male animals.

**Table 1:** Gender dimorphism of heart rate and temperature of mice responses to septic insults.

Parameter	Male		Female	
	Sham	LPS (3 mg/kg)/PepG (0.1 mg/kg)	Sham	LPS (3 mg/kg)/PepG (0.1 mg/kg)
**Number**	6	7	4	8
**Heart Rate (bpm)**	543.33±23.23	486.14±15.07*	569.25±16.44	505.75±12.16*
**Temperature (°C)**	35.38±0.31	30.38±0.87*	35.62±0.46	32.24±0.94*
	**Sham**	**CLP**	**Sham**	**CLP**
**Number**	4	8	4	8
**Heart Rate (bpm)**	537.25±25.76	481.13±11.98*	546.75±10.06	494.25±18.69*
**Temperature (°C)**	35.02±0.52	31.19±0.67*	35.45±0.32	32.08±0.81*
	**Sham**	**LPS (9 mg/kg)/** **PepG (1 mg/kg)**	**Sham**	**LPS (9 mg/kg)/** **PepG (1 mg/kg)**
**Number**	5	11	4	6
**Heart Rate (bpm)**	550.50±26.34	456.72±12.08*	570.75±20.14	448.17±28.53*
**Temperature (°C)**	35.52±0.44	29.16±0.63*	35.90±0.48	29.70±1.03*

Heart rate and temperature were recorded at 18 hours in mice subjected to LPS/PepG co-administration and at 24 hours in mice that underwent CLP. Bpm, beats per minute. Data are expressed as means ± SEM for n number of observations.

*P<0.05 versus the respective sham group,

# *P*<0.05 versus male LPS/PepG or CLP group.

### Gender Dimorphism of Cardiac Dysfunction in Response to CLP-induced Polymicrobial Sepsis

The murine model of CLP with fluid resuscitation and antibiotic treatment offers a clinically relevant model of abdominal polymicrobial human sepsis. Cardiac dysfunction induced by polymicrobial sepsis caused by CLP was only observed in 8 month-old male mice [19]. We sought to confirm the above observed gender difference of cardiac dysfunction in the CLP animal model in 8 month-old male and female mice. Left ventricular function was assessed using echocardiography at 24 hours after CLP or sham surgery. Mice that underwent CLP had a lower body temperature and a lower heart rate in comparison to sham-operated mice (male sham/female sham versus male+CLP/female+CLP; *P*<0.05; Table 1). In sham-operated mice, there was no difference of EF, FS or FAC between male and female mice (*P*>0.05; Figure 1E–H). When compared to sham-treated mice, polymicrobial sepsis induced by CLP caused a significant reduction in EF (*P*<0.05; Figure 1E–F), FS (*P*<0.05; Figure 1E, 1G) and FAC (*P*<0.05; Figure 1E, 1H) in both male and female mice, indicating the development of cardiac dysfunction *in vivo*. However, female mice that underwent CLP exhibited significantly higher EF, FS and FAC in comparison with male mice (*P*<0.05; Figure 1E–H), indicating the cardiac dysfunction induced by CLP was less pronounced in female than in male animals.

### Gender Dimorphism of the Phosphorylation of Akt in the Hearts of Mice Subjected to LPS (3 mg/kg)/PepG (0.1 mg/kg) Co-administration

**Figure 2:**
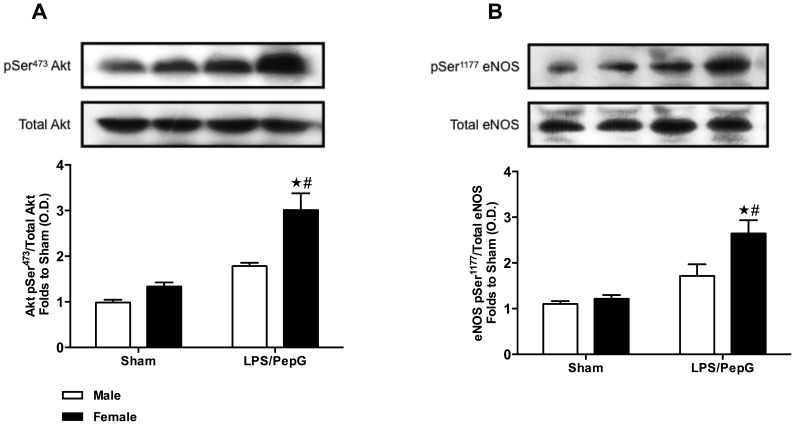
Gender dimorphism of the phosphorylation of Akt and eNOS in the hearts of mice subjected to LPS (3 mg/kg)/PepG (0.1 mg/kg) co-administration. Male or female mice received either LPS (3 mg/kg)/PepG (0.1 mg/kg) or PBS. Signalling events in heart tissue were assessed at 18 hours. Densitometric analysis of the bands is expressed as relative optical density (O.D.) of (**A**) phosphorylated Akt (pSer^473^) corrected for the corresponding total Akt content and normalized using the related sham band; (**B**) phosphorylated eNOS (pSer^1177^), corrected for the corresponding total eNOS content and normalized using the related sham band. Each analysis (**A–B**) is from a single experiment and is representative of three to four separate experiments. Data are expressed as means ± SEM for n number of observations. ★*P*<0.05 versus the respective sham group, #*P*<0.05 versus male LPS/PepG group.

The potential underlying mechanisms behind the observed gender dimorphism of cardiac dysfunction were investigated by semi-quantitative western blot analysis of the mouse heart subjected to LPS/PepG at 18 hours. When compared to male sham-treated mice, female sham-treated mice showed a higher degree of phosphorylation of Akt on Ser^473^ in heart tissue, but these data were not significant (*P*>0.05; Figure 2A). Exposure of male mice to LPS/PepG for 18 hours caused a small and non-significant increase in the phosphorylation of Akt on Ser^473^ (*P*>0.05; Figure 2A). However, exposure of female mice to LPS/PepG for 18 hours induced a significant increase in the phosphorylation of Akt on Ser^473^ compared with either female sham-treated mice or male LPS/PepG-treated mice (*P*<0.05; Figure 2A).

### Gender Dimorphism of the Phosphorylation of eNOS in the Hearts of Mice Subjected to LPS (3 mg/kg)/PepG (0.1 mg/kg) Co-administration

When compared to male sham-treated mice, female sham-treated mice showed a higher degree of phosphorylation of eNOS on Ser^1177^ in heart tissue, but these data were not significant (*P*>0.05; Figure 2B). Exposure of male mice to LPS/PepG for 18 hours caused a small and not significant increase in the phosphorylation of eNOS on Ser^1177^ (*P*>0.05; Figure 2B). However, exposure of female mice to LPS/PepG for 18 hours induced a significant increase in the phosphorylation of eNOS on Ser^1177^ compared with either female sham-treated mice or male LPS/PepG-treated mice (*P*<0.05; Figure 2B).

### Gender Dimorphism of the Phosphorylation of IκBα in the Hearts of Mice Subjected to LPS (3 mg/kg)/PepG (0.1 mg/kg) Co-administration

**Figure 3:**
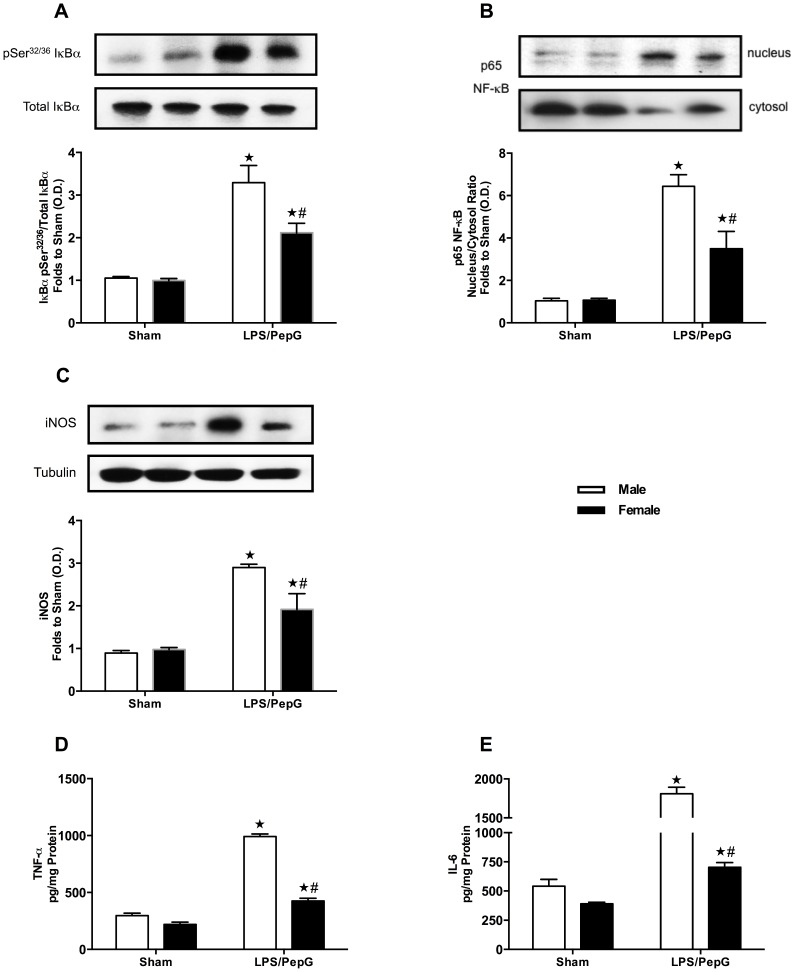
Gender dimorphism of the phosphorylation of IκBα, nuclear translocation of the p65 NF-κB subunit, expression of iNOS, TNF-α and IL-6 in the hearts of mice subjected to LPS (3 mg/kg)/PepG (0.1 mg/kg) co-administration. Male or female mice received either LPS (3 mg/kg)/PepG (0.1 mg/kg) or PBS. Signalling events in heart tissue were assessed at 18 hours. Densitometric analysis of the bands is expressed as relative optical density (O.D.) of (**A**) phosphorylated IκBα (pSer^32/36^) corrected for the corresponding total IκBα content and normalized using the related sham band; (**B**) NF-κB p65 subunit levels in both, cytosolic and nuclear fractions expressed as a nucleus/cytosol ratio normalized using the related sham bands; (**C**) iNOS expression corrected for the corresponding tubulin band, and (**D**) TNF-α expression in heart tissue of mice subjected to LPS/PepG; (**E**) IL-6 expression in heart tissue of mice subjected to LPS/PepG. Each analysis (**A**–**E**) is from a single experiment and is representative of three to four separate experiments. Data are expressed as means ± SEM for n number of observations. ★*P*<0.05 versus the respective sham group, #*P*<0.05 versus male LPS/PepG group.

In sham-treated mice, there was no difference in the phosphorylation of IκBα on Ser^32/36^ between male and female hearts (*P*>0.05; Figure 3A). When compared to sham-treated mice, both male and female mice subjected to LPS/PepG demonstrated significant increases in the phosphorylation of IκBα on Ser^32/36^ in heart tissue (*P*<0.05; Figure 3A). However, the increase in IκBα phosphorylation on Ser^32/36^ caused by LPS/PepG was significantly less pronounced in hearts obtained from female than male mice (*P*<0.05; Figure 3A).

### Gender Dimorphism of Nuclear Translocation of the p65 NF-κB Subunit in the Hearts of Mice Subjected to LPS (3 mg/kg)/PepG (0.1 mg/kg) Co-administration

In sham-treated mice, there was no difference of nuclear translocation of the p65 NF-κB subunit between male and female hearts (*P*>0.05; Figure 3B). When compared to sham-treated mice, both male and female mice subjected to LPS/PepG demonstrated significant increases in the nuclear translocation of the p65 NF-κB subunit in heart tissue (*P*<0.05; Figure 3B). However, female mice subjected to LPS/PepG exhibited a significantly attenuated response in the nuclear translocation of the p65 NF-κB subunit in comparison with male mice (*P*<0.05; Figure 3B), indicating an important role of gender in LPS/PepG induced activation of NF-κB.

### Gender Dimorphism of the Expression of iNOS in the Hearts of Mice Subjected to LPS (3 mg/kg)/PepG (0.1 mg/kg) Co-administration

In sham-treated mice, we detected a faint expression of iNOS protein, but there was no difference of iNOS expression between male and female hearts (*P*>0.05; Figure 3C). When compared to sham-treated mice, LPS/PepG caused significant increases in the expression of iNOS protein in the heart (*P*<0.05; Figure 3C). However, in hearts from female mice subjected to LPS/PepG, the levels of iNOS protein were significantly lower than in hearts from male mice subjected to LPS/PepG (*P*<0.05; Figure 3C).

### Gender Dimorphism of the Expression of TNF-α and IL-6 in the Hearts of Mice Subjected to LPS (3 mg/kg)/PepG (0.1 mg/kg) Co-administration

When compared to male sham-treated mice, female sham-treated mice showed a lower TNF-α and IL-6 expressions in heart tissue, but these data were not significant (*P*>0.05; Figure 3D–E). When compared to sham-treated mice, both male and female mice subjected to LPS/PepG demonstrated significant increases in the expression of TNF-α and IL-6 in heart tissue (*P*<0.05; Figure 3D–E). However, female mice subjected to LPS/PepG exhibited a significantly attenuated response in the expression of TNF-α and IL-6 in comparison with male mice after LPS/PepG challenge (*P*<0.05; Figure 3D–E).

### Gender Dimorphism of Cardiac Dysfunction was Blunted in Response to High Dose of LPS (9 mg/kg)/PepG (1 mg/kg) Co-administration

**Figure 4:**
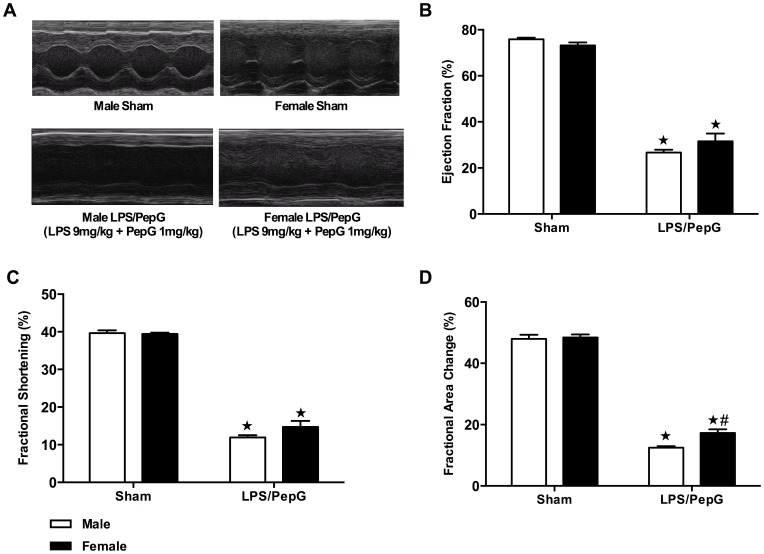
Gender dimorphism of cardiac dysfunction was blunted in response to high dose of LPS (9 mg/kg)/PepG (1 mg/kg) co-administration. Panel **A**–**D**: Male or female mice received either LPS (9 mg/kg)/PepG (1 mg/kg) or PBS intraperitoneally. Cardiac function was assessed at 18 hours. (**A**) Representative M-mode echocardiograms; percentage (%) (**B**) ejection fraction (EF); (**C**) fractional shortening (FS); and (**D**) fractional area of change (FAC). The following groups were studied: Male+vehicle (n = 5); Female+vehicle (n = 4); Male+LPS/PepG (n = 11); Female+LPS/PepG (n = 6). Data are expressed as means ± SEM for n number of observations. ★*P*<0.05 versus the respective sham group, #*P*<0.05 versus male LPS/PepG group.

To further investigate whether the gender dimorphism still exists under increased inflammatory stimulus, left ventricular function was assessed using echocardiography at 18 hours after intraperitoneal injection of LPS (9 mg/kg)/PepG (1 mg/kg) or vehicle. Mice injected with LPS/PepG had a lower body temperature and a lower heart rate in comparison to sham-treated mice (male sham/female sham versus male+LPS/PepG/female+LPS/PepG; *P*<0.05; Table 1). In sham-treated mice, there was no difference in EF, FS or FAC between male and female mice (*P*>0.05; Figure 4A–D). When compared to sham-treated mice, LPS/PepG caused a significant reduction in EF (*P*<0.05; Figure 4A–B), FS (*P*<0.05; Figure 4A, 4C) and FAC (*P*<0.05; Figure 4A, 4D) in both male and female mice, indicating the development of cardiac dysfunction *in vivo*. When compared to male LPS/PepG-treated mice, female mice subjected to LPS/PepG showed a significant increase in FAC (*P*<0.05; Figure 4A, 4D), but this was not significant for EF (*P*>0.05; Figure 4A–B) and FS (*P*>0.05; Figure 4A, 4C), indicating that gender dimorphism of the cardiac dysfunction after septic insult was abrogated by the severe injury induced by high dose of LPS (9 mg/kg)/PepG (1 mg/kg) co-administration.

## Discussion

We describe here for the first time that the myocardial dysfunction caused by LPS/PepG is less pronounced in female than in male mice *in vivo*. This finding is in agreement with the previous reports showing that the cardiac dysfunction caused by myocardial ischemia/reperfusion injury [21], trauma-hemorrhage [22] and burns [23] is also less pronounced in females than in males. Estrogen modulates a number of acute injury-related myocardial responses; specifically estrogen protects the heart against the injury and dysfunction caused by trauma-hemorrhage [24] and ischemia/reperfusion injury (in isolated hearts subjected to global ischemia and in hearts undergoing LAD occlusion *in vivo*) [25], [26]. Although we provide clear evidence that female hearts show less dysfunction than male murine hearts when challenged with LPS/PepG, we wished to confirm this finding by using a more clinically relevant model of polymicrobial sepsis with antibiotic therapy and fluid-resuscitation caused by CLP in middle-aged mice (8 month-old) [18], [19]. The age of mice was selected based on the knowledge that 8 month-old female C57BL/6 mice are pre-ovarian failure and still have an active estrus cycle [27]. Most notably, we demonstrate here that the cardiac function in female mice subjected to polymicrobial sepsis induced by CLP was significantly less pronounced than the cardiac dysfunction observed in male mice. Taken together, these findings indicate that the hearts of young or older female mice exhibit less cardiac dysfunction in response to polymicrobial sepsis or co-administration of LPS/PepG.

To obtain a better insight into the mechanisms underlying the observed gender dimorphism of the cardiac response to sepsis, we investigated the phosphorylation of Akt, eNOS and IκBα, nuclear translocation of NF-κB subunit p65, iNOS expression, as well as TNF-α and IL-6 expression in murine hearts; When compared to the hearts of male mice subjected to LPS/PepG, hearts of female mice subjected to LPS/PepG showed i) profound increases in phosphorylation of Akt and eNOS; ii) reductions in phosphorylation of IκBα and nuclear translocation of the NF-κB subunit p65, iii) reduced expression of the pro-inflammatory cytokines TNF-α and IL-6, and iv) reduced expression of iNOS.

Akt is a member of the phosphoinositide 3-kinases (PI3K) signal transduction enzyme family, activation of which protects the heart against injury [28], [29]. Here we demonstrate that co-administration of LPS/PepG to female rather than male mice leads to a greater increase in Akt-phosphorylation and, hence, activity in the heart of female animals. Indeed, a greater increase in cardiac Akt phosphorylation in female when compared to male hearts also accounts for the reduced cardiac injury caused by ischemia-reperfusion in female mice. Most notably, when the Akt-pathway is blocked, the degree of cardiac injury in male and female mice was identical. Thus, activation of cardiac Akt (presumably by estradiol) protects female hearts against cardiac injury and dysfunction [30]. Estradiol activates cardiac Akt, which in turn also leads to a reduction in the cardiac dysfunction caused by trauma-hemorrhage [24], [29]. Blockade of the Akt pathway also abrogated the salutary effects of estradiol on cardiac function following trauma-hemorrhage [24]. Moreover, activation of Akt mediates the inhibition by estradiol of the TNF-α expression and NF-κB activation caused by LPS in cardiomyocytes [31]. In the present study, we found a small increase in cardiac Akt activity in female than in male sham hearts. In line with this finding, one previous study showed that young women possess higher levels of Akt in the myocardium compared to comparably aged men or postmenopausal women, and that sexually mature female mice have elevated Akt kinase activity in nuclear extracts of hearts than male mice [32]. The hypothesis that cardiac Akt activity is modulated by estrogen is also supported by the finding that the Akt activation in cardiomyocytes was reduced in ovariectomized rats [33]. In addition, activation of the PI3K/Akt signalling cascade by estrogen was observed in rat cardiomyocytes [29]. A few studies have been conducted to explain the exact mechanism by which estrogen induces Akt activation. Estrogen receptor α has been shown to bind with the p85 α regulatory subunit of PI3K in a ligand-dependent manner in human endothelial cells; increased estrogen receptor associated PI3K activity induced by estrogen leads to the activation of Akt and eNOS in human endothelial cells [34]. Another study has shown that the direct interaction between estrogen receptor and the PI3K regulatory subunit p85 in a time-dependent manner was consistent with the temporal profile for Akt phosphorylation in neurons [35]. Additionally, in cardiomyocytes, estrogen stimulated Akt activation and prevented DNA fragmentation [29]. Thus, we propose that the higher cardiac activation of Akt in female mice importantly contributes to the improvement in cardiac dysfunction in sepsis.

Activation of Akt is known to modulate eNOS activity through phosphorylation of eNOS at Ser^1177^ [36], [37]. Indeed, the present study reported an increase in eNOS phosphorylation in female than in male hearts, which was correlated with the expression pattern of Akt. Augmentation of eNOS activity was shown to decrease sepsis-related increases in neutrophil-endothelial cell interaction and potentially maintain microvascular patency in sepsis [38]. There is good evidence that estrogen modulates activation of eNOS. Estrogen receptor α has been implicated in increased PI3K/Akt and eNOS activation induced by estrogen in human endothelial cells [34]. Another study demonstrated that estrogen stimulation of the eNOS promoter was mediated via increased activity of the transcription factor Sp1 (which is essential for the activity of the human eNOS promoter) [39]. Moreover, estradiol treatment in guinea pigs increased eNOS mRNA in skeletal muscle, suggesting an increase in eNOS activity [40]. In line with these findings, data from the present study indicate that less vulnerability of female hearts to sepsis may be mediated in part by an increased activity of eNOS, secondary to the activation of PI3K/Akt pathway.

NF-κB controls the transcription of a large number of genes, particularly those involved in inflammatory and acute stress responses, such as cytokines, chemokines, cell adhesion molecules, apoptotic factors, and other mediators [41]. IκBα inactivates NF-κB by masking the nuclear localization signals of the NF-κB proteins and by sequestering NF-κB as an inactive complex in the cytoplasm [41], [42]. Phosphorylation of IκBα by IκB kinase (IKK) leads to the dissociation of IκBα from NF-κB, which liberates NF-κB to enter the nucleus and activates the expression of NF-κB target genes [41]. Up-regulation of NF-κB has been linked to the development of myocardial dysfunction following the onset of sepsis [19], [43]. Inhibition of NF-κB activation results in improved myocardial function after septic challenge [18]. Additionally, the dimer of estrogen and its receptor can bind to NF-κB in osteoblasts following IL-1β exposure, further, NF-κB is proved to be one of the targets for estrogen receptor, resulting in reduced IL-6 promoter activity [44]. In murine splenic macrophages, estradiol inhibited TNF-α and IL-6 production was associated with a decreased LPS-induced NF-κB-binding activity [44]. Thus, our present results indicate that less myocardial dysfunction in females subjected to LPS/PepG could be importantly due to the decreased activation of NF-κB (secondary to the reduced activation of IκBα and, hence, nuclear translocation) in murine hearts. Activation of NF-κB may also mediate myocardial dysfunction through induction of expression of its target gene iNOS, which plays an important role in sepsis-related hypotension and impaired left ventricular function [45], [46]. Indeed, in the present study, iNOS expression was increased in male hearts, which correlates with their exacerbated cardiac dysfunction under septic insult.

In addition to causing the expression of iNOS, NF-κB activation also leads to a pronounced increase in production of inflammatory mediators such as TNF-α and IL-6 [47]. In turn, TNF also activates NF-κB through TNF-receptor-associated factors, this increases cytokine production, thus forming a feed-forward mechanism and amplifying the inflammatory reaction [48]. There is good evidence that those inflammatory cytokines play a significant role in the pathogenesis of sepsis-induced cardiac dysfunction [49], [50]. Moreover, clinical studies showed that stimulation of healthy females with LPS or LTA led to lower TNF-α and IL-6 levels in blood than males [10]. Female patients with sepsis had a higher survival rate, which was correlated with lower TNF-α and higher IL-10 levels [9], while male trauma-patients showed higher IL-6 level than females [11]. In experimental studies, cardiomyocyte TNF-α and IL-6 release was markedly lower in female than male rats following burn injury [23]. In addition, female hearts expressed less myocardial TNF-α in isolated hearts subjected to ischemia/reperfusion injury [21] or LPS treatment [51]. Others have suggested that elevated plasma TNF-α and IL-6 induced by trauma-hemorrhage was prevented by estradiol treatment in rats [24], [52]. Consistent with these findings, in our study, female mice, which had better cardiac function following septic insult, expressed less myocardial TNF-α and IL-6 than male mice subjected to LPS/PepG co-administration.

Our study demonstrated that the gender dimorphism of cardiac dysfunction in response to septic insults was abolished by the severe injury induced by high dose of LPS (9 mg/kg)/PepG (1 mg/kg) co-administration. This is in line with a report that the inflammatory cytokine response differed more strongly between blood from men and women after low-concentration of LPS stimulation compared with a higher stimulus concentration [10]. Population-based studies on sex dimorphism in mortality after sepsis showed inconsistent results. Some studies reported increased mortality in males [7], [8], while other studies demonstrated mortality from severe sepsis/sepsis was not affected by gender [53], [54]. The inconsistency may have resulted from multiple factors such as pre-existing co-morbidities. More importantly, our observations of gender dimorphism in cardiac dysfunction responses to different severities of injury may partially explain the conflicting clinical data.

It could be argued that the present study did not provide information about proestrus/estrus or diestrus state of estrus cycle in female mice subjected to septic insults. In this regard, a recent study showed that female mice with CLP survived better than male mice that underwent CLP, but the higher survival in females did not correspond to any specific estrus phase [55]. Furthermore, it has been demonstrated vaginal cytology does not reflect changes of circulating estrogens in females and that the estrus cycle cannot be predicted by vaginal smears [56]. Moreover, we did not notice a lot of variations in data obtained from female mice in our study. Therefore, estrus cycle phases were not monitored in this study.

## Conclusion

Our findings provide for the first time a very clear indication of a gender dimorphism in the sepsis-induced cardiac dysfunction *in vivo* and we have shown that female mice have less cardiac dysfunction than male mice subjected to either co-administration of LPS/PepG in young mice or CLP in older mice. We report here that female hearts subjected to sepsis have a greater activation of Akt/eNOS, and less activation of NF-κB, which in turn results in reduced expression of the proinflammaroy cytokines TNF-α and IL-6 as well as iNOS. We propose that the above pro-survival and anti-inflammatory signalling events contribute to the reduced cardiac dysfunction in female mice with sepsis.
